# Vector analysis of low to moderate astigmatism with small incision lenticule extraction (SMILE): results of a 1-year follow-up

**DOI:** 10.1186/1471-2415-15-8

**Published:** 2015-01-24

**Authors:** Jiamei Zhang, Yan Wang, Wenjing Wu, Lulu Xu, Xiaojing Li, Rui Dou

**Affiliations:** Tianjin Eye Hospital & Eye Institute, Tianjin Key Laboratory of Ophthalmology and Visual Science, Tianjin Medical University, No.4 Gansu Rd, Heping District, Tianjin, 300020 China

**Keywords:** Vector analysis, Astigmatism, Femtosecond laser, Small incision lenticule extraction, Refractive surgery, Long-term

## Abstract

**Background:**

To evaluate the refractive outcomes for the correction of low to moderate astigmatism up to 1 year following small incision lenticule extraction (SMILE) surgery.

**Methods:**

This retrospective study enrolled 98 eyes from 98 patients who underwent SMILE surgery for the correction of myopia and astigmatism. Only right eyes were included in this study to avoid the bias of orientation errors. The vector method was used to analyze the outcomes of astigmatism at 1 month, 6 months and 12 months after the procedure, including the double-angle plots, correction index (CI), index of success (IOS), angle of error (AofE) and magnitude of error (MofE). The effectiveness, safety, stability and predictability were also investigated during the 12-month follow-up.

**Results:**

The preoperative cylinder ranged from -2.75 D to -0.25 D (average of -0.90 ± 0.68 D), and the mean postoperative cylinder values were -0.24 ± 0.29 D, -0.24 ± 0.29 D, and -0.20 ± 0.27 D at 1 month, 6 months, and 12 months, respectively. The mean astigmatism in vector form was -0.14 D × 27.19° at 1 month, -0.13 D × 27.29° at 6 months, and -0.10 D × 28.63° at 12 months after surgery. The CI was 1.00 ± 0.32 and IOS was 0.29 ± 0.44 at the 12-month follow-up. Significant negative correlations were found between the CI and absolute target induced astigmatism (TIA) value, and positive correlations were found between the IOS and absolute AofE value (P < 0.05). The MofE was limited within ±1.00 D at the 12-month follow-up. Fifty-six eyes (57.1%) gained one line in corrected distance visual acuity (CDVA) and five eyes (5.1%) gained two lines. There were no significant differences observed in the refractive outcomes among time points.

**Conclusions:**

SMILE surgery was effective and safe in correcting low to moderate astigmatism, and stable refractive outcomes were observed at the long-term follow-up. The undercorrection of astigmatism could possibly be influenced by attempted astigmatism correction preoperatively, the axis rotation during the surgery or wound healing postoperatively. This study suggested that nomograms should be adjusted in correcting astigmatism with SMILE surgery.

## Background

Since small incision lenticule extraction (SMILE) surgery was first published in 2011 by Shah [1] and Sekundo [2], it has gained great interest among refractive surgeons for its flapless feature and all-in-one femtosecond laser procedure. However, compared to the previous laser in-situ keratomileusis (LASIK) procedure, the lenticule of SMILE surgery is created by the femtosecond laser without eye-tracking, instead of photoablation performed by excimer laser, and the accuracy of the axis correction is highly influenced by the alignment between the center of the ablated zone and the center of the pupil. Therefore, there has been some controversy in treating astigmatism with SMILE surgery. Refractive results have previously been analyzed in several studies [1–6]; however, few have focused on correcting astigmatism, especially in the vector method. Recently, a study on correcting astigmatism following femtosecond lenticule extraction (FLEx) was investigated by Kunert et al. [7] in vector form, which had a promising result. Therefore, the vector analysis of SMILE surgery might play an important role in the evaluation of astigmatism.

In the latest study, Ivarsen et al. [8] reported the correction of low to high degrees of myopic astigmatism in a polar value at the 3-month follow-up. However, the magnitude and direction of astigmatism are not constant due to the wound healing process after corneal refractive surgery. To the best of our knowledge, long-term results in correcting low to moderate astigmatism have not yet been evaluated. This study sought to analyze the correction of astigmatism according to the Alpins method of astigmatism analysis [9], and focused on a longer follow-up after SMILE surgery.

## Methods

### Patients

A nonrandomized and retrospective study was performed on 98 patients (98 right eyes) who underwent SMILE surgery at Tianjin Eye Hospital, Tianjin Medical University, China, for the correction of myopic astigmatism between August 2011 and December 2013. The study was approved by the Ethics Committee of Tianjin Eye Hospital and adhered to the tenets of the Declaration of Helsinki. The inclusion criteria were a minimum age of 18 years, stable refraction for at least 1 year, a clear cornea without opacity, central corneal thickness more than 500 μm, calculated residual stroma more than 250 μm, intraocular pressure (IOP) less than 21 mmHg and no other ocular conditions, except myopic astigmatism. The exclusion criteria were keratoconus or suspicious keratoconus and systemic diseases. The patients who wore soft contact lenses were instructed to stop wearing them for at least 2 weeks, and rigid contact lenses were discontinued for at least 4 weeks.

All patients were required to undergo a thorough eye examination preoperatively and at 1 day, 1 week, 1 month, 3 months, 6 months and 12 months postoperatively. The routine examinations included the assessment of uncorrected distance visual acuity (UCVA), corrected distance visual acuity (CDVA), manifest and cycloplegic refraction, slit-lamp microscopy, dilated funduscopy, pupil size, corneal topographic measurement (Pentacam-HR, Oculus GmbH, Wetzlar, Germany) and non-contact tonometry (Topcon-CT80, Topcon, Tokyo, Japan). Informed consent forms were signed before SMILE surgery in all patients. All patients were targeted to achieve emmetropia and completed the observation up to 12 months. Experienced optometrists achieved the manifest refraction. Because the cornea had slight edema at the early stage after surgery, we adopted the refraction data for analysis since the first month postoperatively. In this study, the refraction data analyzed at 1 month were considered early phase, the data at 6 months were medium phase and the data at 12 months were long-term phase after SMILE surgery.

### Surgical procedure

The same experienced surgeon performed the SMILE surgery for all patients. After the eyes were surface anesthetized with oxybuprocaine eye drops (Benoxil, Santen, Inc., Japan) 3 times preoperatively, patients were told to fix on the target light so that suction could be initiated. The surgeon confirmed that the center of the ablated zone was aligned with the center of the pupil; after that, the surgery was performed using a 500-kHz Visu Max femtosecond laser (Carl Zeiss, Meditec AG, Jena, Germany) with laser energy of approximately to 170 nJ. Each spot was spaced 1.5 μm apart and created a photodisruption in the stroma. The posterior surface was first scanned from the periphery to the center; then, the anterior was scanned from the center to the periphery. The refractive lenticule diameter was performed in 6–6.5 mm with a transition zone of 0.1 mm. The cap thickness was 110 μm. An average 3.73 mm side-cut incision (range from 2 to 5 mm) was created at the 12 o’clock position of the cornea. Once the femtosecond laser cutting procedure was finished, the suction switched off and the lenticule was extracted from the cornea. The detailed surgical procedure was previously described [1].

After the surgery, 0.3% ofloxacin (Tarivid, Santen, Inc., Japan) eye drops were topically used 4 times daily for 3 days and 0.1% fluorometholone (Flumetholon, Santen, Inc., Japan) eye drops were used 4 times daily for the first 2 weeks and then reduced to 3, 2, 1 times every 2 weeks.

### Vector method

The evaluation of astigmatism was mainly based on the definitions and formulas given by Alpins [9–11]. Because there was some degree of a mirror symmetric effect in the axes of astigmatism between the right and left eyes [12], they were not supposed to be analyzed in the same direction. To avoid the bias of orientation errors, only right eyes were included in this study. Furthermore, the refraction data were analyzed in the negative-cylinder form, and Holladay [13] noted that the negative sign indicated a reduction in the dioptric power in myopic correction. However, deviating from Holladay [13], we used the axis form to describe the direction of astigmatism rather than the power notation; therefore, the right side of the X-axis would be with-the-rule astigmatism and the left side of the X-axis would be against-the-rule in the double-angle plots. The manifest refraction data were not converted to the corneal plane, and the vertex distance was set at 12 mm.

As suggested by Alpins [9], the target induced astigmatism (TIA) was defined as the difference between the preoperative astigmatism and target astigmatism in vector operation. The TIA value was equal to the preoperative cylinder in this study because the target refraction was emmetropia. Accordingly, the difference vector (DV) was equal to the postoperative cylinder. The surgically induced astigmatism (SIA) was defined as the vector difference between the preoperative cylinder and postoperative cylinder as the actual correction achieved. The index of success (IOS) was calculated by the magnitude of DV over the magnitude of TIA, which indicated that the residual astigmatism still existed (IOS = |DV|/|TIA|). The correction index (CI) was given as the proportion of the magnitude of SIA and TIA that showed that the intended correction was not successfully treated (CI = |SIA|/|TIA|). The angle of error (AofE) was measured in the half angular between the SIA and TIA vectors, which indicated whether the treatment was applied at correct axis. The magnitude of error (MofE) was defined as the arithmetic difference between the magnitude of the TIA and the SIA (MofE = |SIA|-|TIA|).

### Statistical analysis

All data were collected and calculated using Excel 2007 (Microsoft Corp., Redmond, WA, USA), and descriptive statistical analyses were performed using SPSS 17.0 (SPSS Inc., Chicago, IL, USA). The double-angle plots were drawn by Sigma Plot 10.0 (Systat Software, San Jose, CA, USA). Because the data did not show a normal distribution, nonparametric analysis was used in this study. The Friedman test was applied to compare variables among each follow-up. The Spearman rank correlation coefficient and linear scatter plots were used to assess the association between variables. A value of P less than 0.05 was considered statistically significant. In addition, the refractive outcomes were analyzed according to Waring et al. [14] at the 12-month follow-up. All data are described as the mean and standard deviation (Mean ± SD).

## Results

The study included 98 eyes from 98 patients. All patients had accomplished the follow-up at 1 month, 6 months and 12 months without anyone dropping out, and none of the subjects were retreated during this follow-up period. The basic preoperative characteristics of these patients were summarized, and emmetropia was attempted in all patients (Table 1).

**Table 1:** **Basic preoperative characteristics of 98 patients**

Parameter	Value
Gender (n)	
Female	52
Male	46
Age (y)	
Mean ± SD	22.82 ± 4.65
Range	18,39
Sphere (D)	
Mean ± SD	-5.10 ± 1.48
Range	-8.50,0
Cylinder (D)	
Mean ± SD	-0.90 ± 0.68
Range	-2.75,–0.25
SE* (D)	
Mean ± SD	-5.55 ± 1.39
Range	-9.00,–1.25

*SE = Spherical equivalent.

### Vector analyses

**Figure 1:**
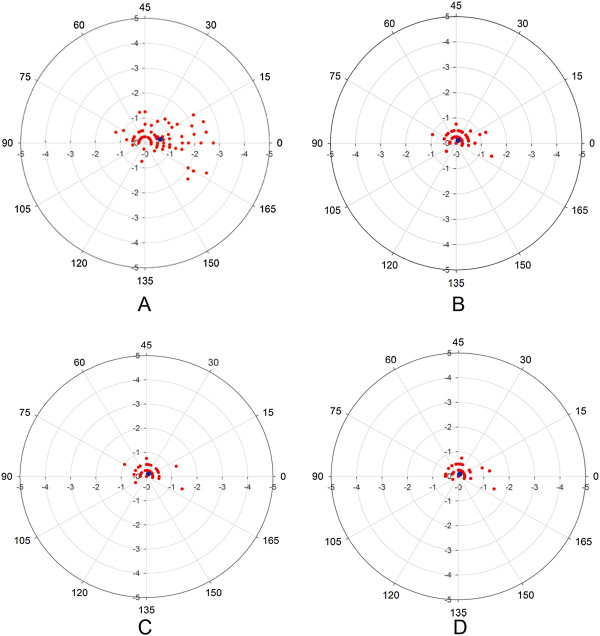
**The double-angle plots of the astigmatism.** The preoperative **TIA (A)** and postoperative **DV** at 1 month **(B)**, 6 months **(C)** and 12 months **(D)**. The vector of astigmatism is represented by the red spots and the blue spot indicates the centroid value of each plotted vector.

The double-angle plots demonstrated the preoperative TIA and postoperative DV at each follow-up of 98 eyes (Figure 1). The centroid coordinates (x, y) of TIA were (0.61 ± 0.83, 0.15 ± 0.44), which indicated that the average astigmatism was with-the-rule before surgery. At each follow-up, the centroid coordinates (x, y) of DV were (0.08 ± 0.30, 0.11 ± 0.19) at 1 month, (0.08 ± 0.31, 0.11 ± 0.18) at 6 months, and (0.06 ± 0.27, 0.09 ± 0.17) at 12 months. The mean astigmatism in vector form was -0.63 D × 6.68° preoperatively and -0.14 D × 27.19° at 1 month, -0.13 D × 27.29° at 6 months, and -0.10 D × 28.63° at 12 months after surgery. These results indicated a reduction in the cylinder value and the axis of astigmatism toward counterclockwise with long-term observation.

**Figure 2:**
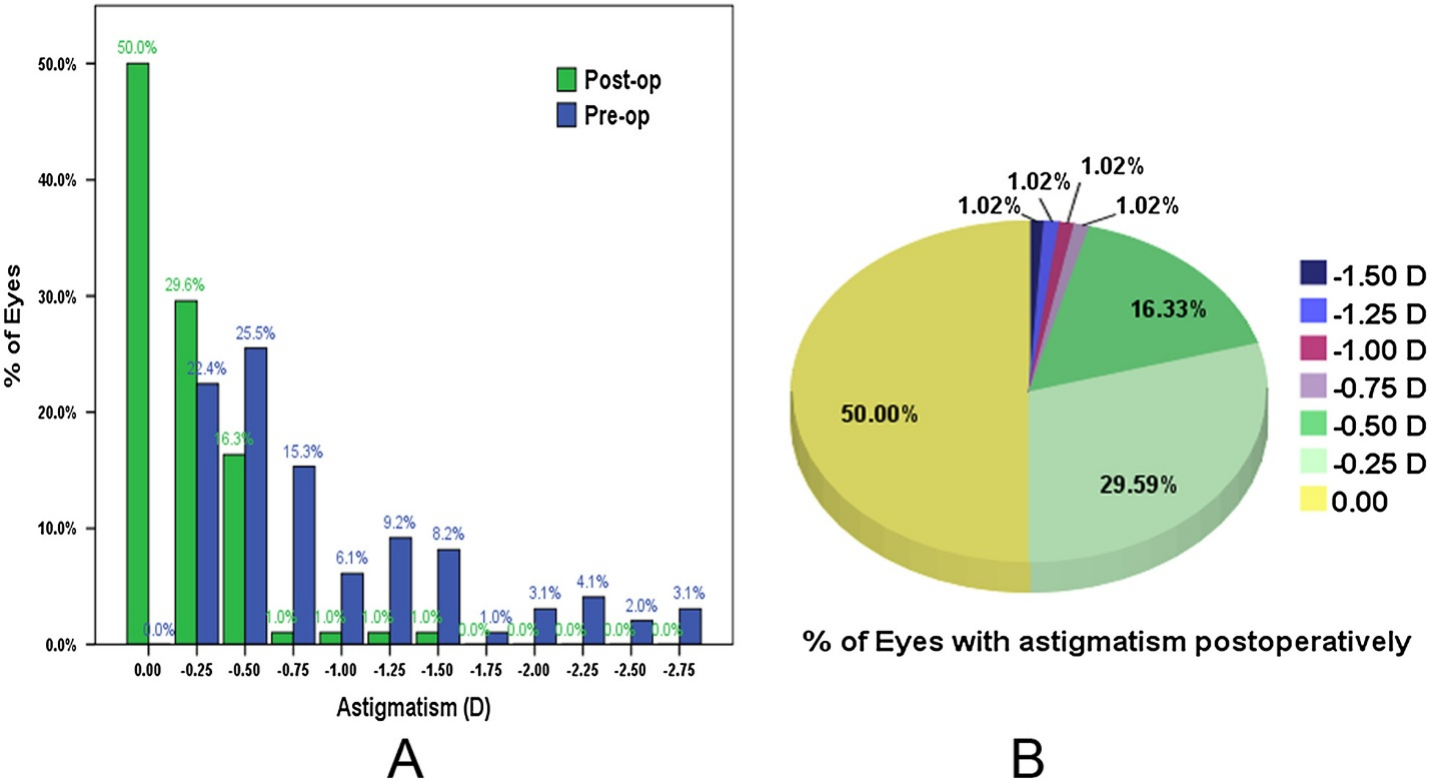
**The effectiveness of SMILE surgery.** Postoperative cylinder compared with the preoperative cylinder **(A)** and pie chart of the cylinder at the 12-month follow-up **(B)**.

Ninety-four eyes (95.9%) had a postoperative negative-cylinder value within -0.50 D at the 12-month follow-up (Table 2). These results indicated a counterclockwise change toward the oblique axis, especially at a low cylinder value, which corresponded to the location of scatters in double-angle plots. The cylinder values at the 12-month follow-up were demonstrated in a pie chart and compared with the preoperative cylinder (Figure 2).

**Table 2:** **Postoperative astigmatism at the 12-month follow-up of 98 eyes**

Postoperative	Absolute shift in axis ^†^ (n)				
cylinder (D)	≤15°	>15°to ≤ 30°	>30°to ≤ 45°	>45°	Total
0*	49	-	-	-	49
>0.00 to ≤ -0.50	16	11	7	11	45
> - 0.50 to ≤ -1.00	1	0	1	0	2
> - 1.00	1	1	0	0	2
Total	67	12	8	11	98

^†^Axis shift is determined from the postoperative to preoperative cylinder axis.

*Shifts are defined as zero for eyes with zero residual cylinder magnitude.

The CI and IOS decreased with the degree of preoperative cylinder increased (Table 3, Table 4). With extended time, the CI had a tendency to increase and the IOS had a tendency to decrease. However, no statistical significances were observed in the total CI and total IOS between each follow-up (*X*^2^ = 2.235, P = .327 of CI and *X*^2^ = 5.409, P = .067 of IOS, Friedman test). Significant negative correlations were observed between the CI and absolute TIA value at each follow-up (r = -0.461, P = .000 at 1 month, r = -0.311, P = .002 at 6 months, r = -0.321, P = .001 at 12 months, Spearman rank correlation).

**Table 3:** **Correction index (CI) at each follow-up of 98 eyes (Mean ± SD)**

CI	Preoperative cylinder (D)				
	>0 to ≤ -0.50	> - 0.50 to ≤ -1.00	> - 1.00 to ≤ -2.00	> - 2.00 to ≤ -3.00	Total
	(n = 47)	(n = 21)	(n = 21)	(n = 9)	(n = 98)
1 month	1.03 ± 0.27	0.96 ± 0.24	0.87 ± 0.18	0.83 ± 0.16	0.96 ± 0.25
6 months	1.03 ± 0.40	0.95 ± 0.31	0.91 ± 0.19	0.85 ± 0.17	0.97 ± 0.33
12 months	1.05 ± 0.41	1.03 ± 0.24	0.91 ± 0.19	0.87 ± 0.15	1.00 ± 0.32

**Table 4:** **Index of success (IOS) at each follow-up of 98 eyes (Mean ± SD)**

IOS	Preoperative cylinder (D)				
	>0 to ≤ -0.50	> - 0.50 to ≤ -1.00	> - 1.00 to ≤ -2.00	> - 2.00 to ≤ -3.00	Total
	(n = 47)	(n = 21)	(n = 21)	(n = 9)	(n = 98)
1 month	0.81 ± 2.91	0.27 ± 0.29	0.26 ± 0.19	0.22 ± 0.22	0.52 ± 2.03
6 months	0.43 ± 0.54	0.32 ± 0.29	0.23 ± 0.22	0.19 ± 0.23	0.34 ± 0.42
12 months	0.39 ± 0.57	0.20 ± 0.24	0.20 ± 0.22	0.17 ± 0.21	0.29 ± 0.44

The value of AofE was usually high when the astigmatism was low, which was because the measurement error tended to be relatively obvious (Table 5). A negative value of AofE indicated that the SIA was clockwise from the TIA, and a positive value indicated a counterclockwise rotation from its intended axis. The absolute value of AofE deviated from the intended direction. There were strong, statistically significant correlations between the IOS and absolute AofE value (r = 0.938, P = .000 at 1 month, r = 0.951, P = .000 at 6 months, r = 0.963, P = .000 at 12 months, Spearman rank correlation). Few patients had a |AofE| more than 15°.

**Table 5:** **Angle of error (AofE) at the 12-month follow-up of 97 eyes**

Preoperative cylinder (D)	AofE	|AofE|	|AofE| ≤ 15°	AofE > +15	AofE < -15°
	(Mean ± SD)	(Mean ± SD)	(n)	(n)	(n)
>0 to ≤ -0.50	-3.44 ± 15.69	8.43 ± 13.63	39	1	6
(n = 46)					
> - 0.50 to ≤ -1.00	-3.27 ± 5.13	3.84 ± 4.69	21	0	0
(n = 21)					
> - 1.00 to ≤ -2.00	-3.44 ± 6.93	4.31 ± 6.40	20	1	0
(n = 21)					
> - 2.00 to ≤ -3.00	0.53 ± 6.86	3.42 ± 5.85	8	1	0
(n = 9)					
Total	-3.04 ± 11.67	6.08 ± 10.40	88	3	6
(n = 97)					

Undercorrection was indicated when the value of MofE was negative, and vice versa, a positive MofE indicated overcorrection of the astigmatism (Table 6). Both MofE and CI could indicate whether the astigmatism was fully corrected from its intended magnitude. When the preoperative cylinder was more than -1.00 D, the value of MofE exactly indicated the undercorrection.

**Table 6:** **Magnitude of error (MofE) at the 12-month follow-up of 98 eyes**

Preoperative cylinder (D)	MofE	|MofE|	MofE ≤ ±1.00D	MofE ≤ ±0.50D
	(Mean ± SD)	(Mean ± SD)	(n)	(n)
>0 to ≤ -0.50	0.01 ± 0.12	0.07 ± 0.10	47	47
(n = 47)				
> - 0.50 to ≤ -1.00	0.02 ± 0.19	0.10 ± 0.16	21	21
(n = 21)				
> - 1.00 to ≤ -2.00	-0.13 ± 0.28	0.18 ± 0.24	21	20
(n = 21)				
> - 2.00 to ≤ -3.00	-0.32 ± 0.35	0.32 ± 0.35	9	7
(n = 9)				
Total	-0.05 ± 0.23	0.12 ± 0.20	98	95
(n = 98)				

### Effectiveness and safety

**Figure 3:**
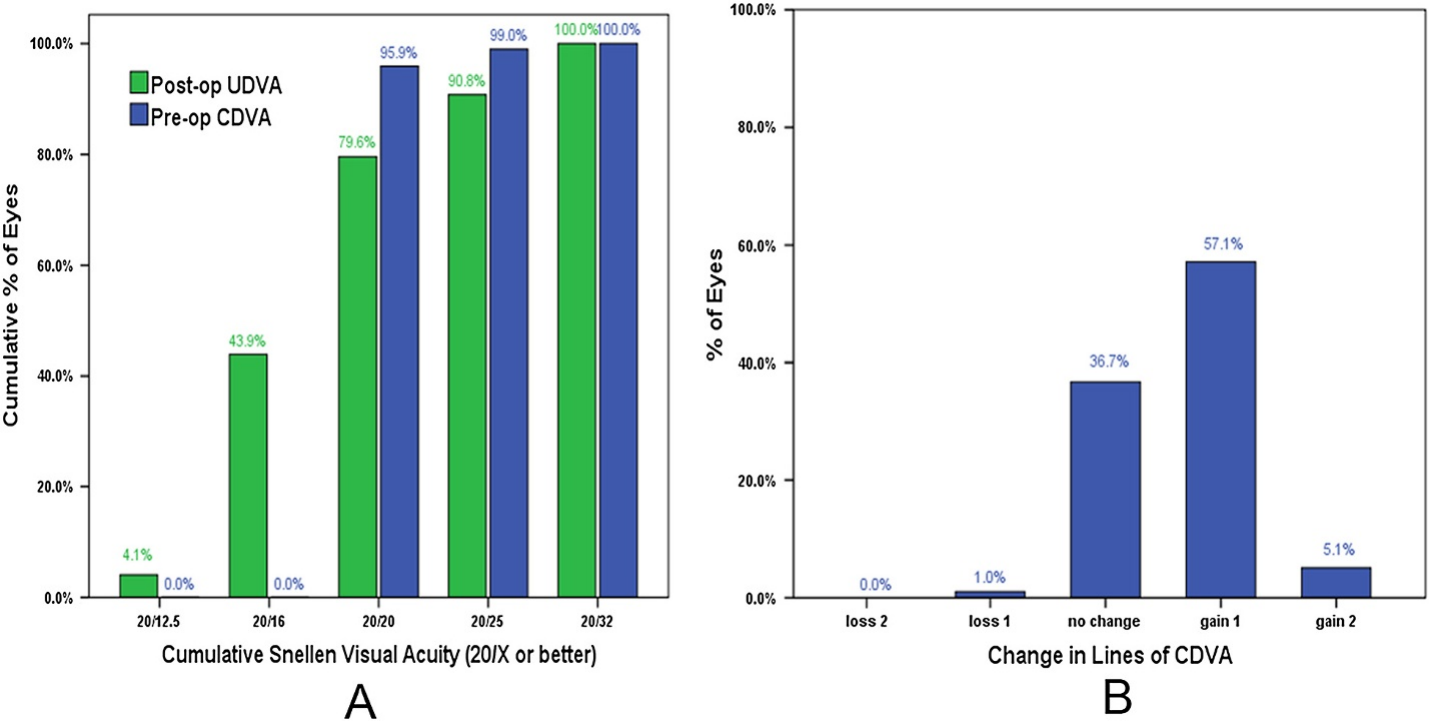
**The effectiveness and safety of SMILE surgery.** The UDVA at the 12-month follow-up compared with the preoperative CDVA in cumulative of eyes **(A)** and change in lines of CDVA at 12 months after surgery **(B)**.

Preoperatively, the median of UDVA was 20/200. Eighty-nine eyes (90.8%) had a UDVA of 20/25 or better and seventy-eight eyes (79.6%) had a UDVA of 20/20 or better at the 12-month follow-up. Nearly half of the eyes (43.9%) achieved a UDVA more than 20/20, which was beyond their expectations, and a large number of patients were quite satisfied with their visual acuity. Thirty-six eyes (36.7%) had an unchanged CDVA, and fifty-six eyes (57.1%) gained one line. Only one eye (1.0%) lost one line, and none of the eyes lost two or more lines (Figure 3). None of the patients had corneal complications, except for three who complained of dry eye at the 12-month follow-up.

### Stability

**Figure 4:**
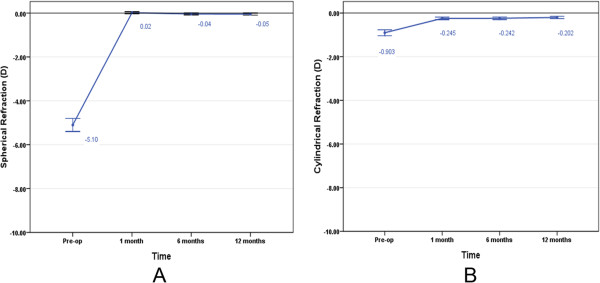
**The stability of SMILE surgery.** The average of spherical refraction **(A)** and cylindrical refraction **(B)** changed with time. The mean value is demonstrated and the error bar illustrates the 95% confidence interval (CI).

There were no significant differences between each follow-up of spherical refraction and cylindrical refraction, indicating that the refractive outcomes stabilized after SMILE surgery (*X*^2^ = 5.249, P = .072 of sphere and *X*^2^ = 4.986, P = .083 of cylinder, Friedman test). The mean postoperative sphere values were 0.02 ± 0.22 D, -0.04 ± 0.21 D, and -0.05 ± 0.24 D at 1 month, 6 months and 12 months, respectively (Figure 4A). The mean postoperative cylinder values were -0.24 ± 0.29 D, -0.24 ± 0.29 D, and -0.20 ± 0.27 D at 1 month, 6 months and 12 months, respectively (Figure 4).

### Predictability

**Figure 5:**
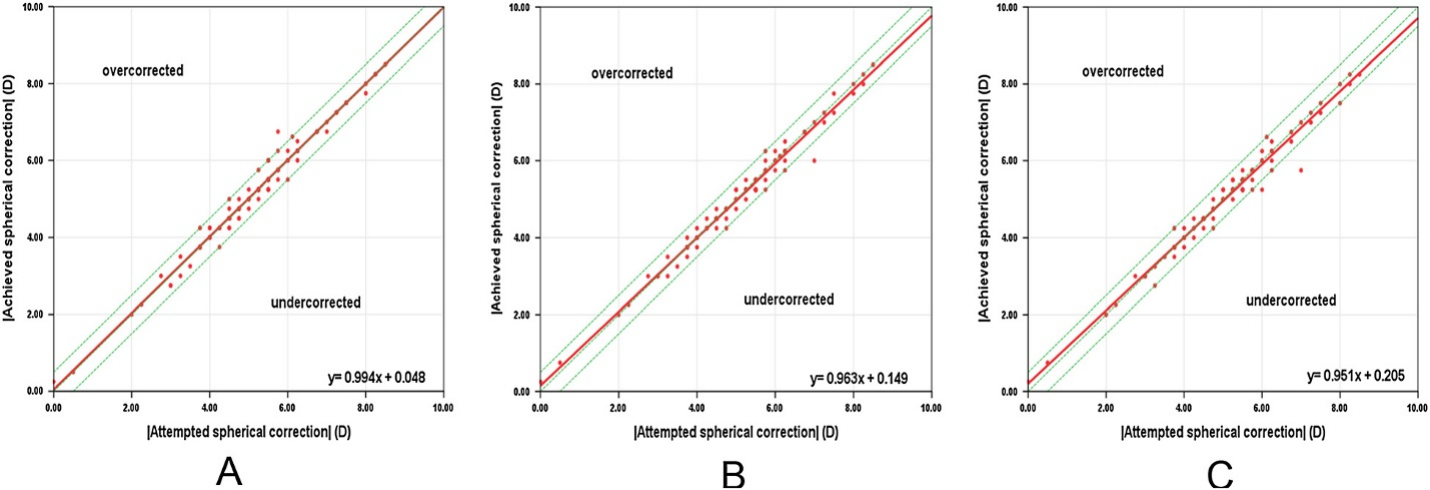
**The predictability of spherical refraction with SMILE surgery.** The attempted versus achieved spherical correction in the absolute value with SMILE surgery at 1 month **(A)**, 6 months **(B)** and 12 months **(C)**. Above the green dashed line in the middle is overcorrection and below is undercorrection. The red solid line indicates the outcome of linear regression analysis.

**Figure 6:**
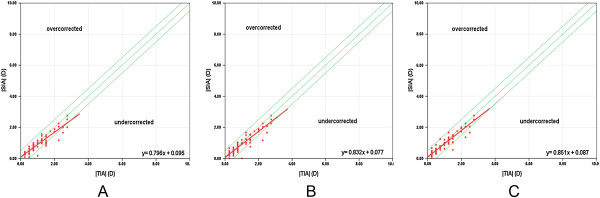
**The predictability of cylindrical refraction with SMILE surgery.** The absolute TIA versus SIA with SMILE surgery at 1 month **(A)**, 6 months **(B)** and 12 months **(C)**. Above the green dashed line in middle is overcorrection and below is undercorrection. The red solid line indicates the outcome of linear regression analysis.

Regarding the spherical diopter after SMILE surgery, the spherical refraction was almost fully corrected (Figure 5). There were strong correlations between the attempted spherical correction and achieved spherical correction in the absolute value at each follow-up (r = 0.984, P = .000 at 1 month, r = 0.984, P = .000 at 6 months, r = 0.980, P = .000 at 12 months, Spearman rank correlation). Regarding the cylindrical refraction, there was a tendency toward undercorrection as the preoperative astigmatism increased (Figure 6). The absolute values of the SIA and TIA were highly correlated at each follow-up (r = 0.925, P = .000 at 1 month, r = 0.911, P = .000 at 6 months, r = 0.944, P = .000 at 12 months, Spearman rank correlation).

## Discussion

The evaluation of the astigmatic correction has important clinical significance, especially in refractive surgery. SMILE surgery, which is recommended as the most advanced technique, has demonstrated promising results in correcting refractive error [1–6]. The surgery is performed by making a refractive lenticule within the corneal stroma and then extracting the lenticule via a small incision rather than the creation of a corneal flap. Compared to the FLEx or FS-LASIK method, the flapless feature theoretically induces less injury to the cornea [15–17] and maintains better corneal morphology [18] and biomechanical effects [19]. The aim of the current study is to investigate the long-term effects of correcting low to moderate astigmatism following SMILE surgery, which has never been reported before.

The spherical equivalent (SE) has generally been used to evaluate the refractive outcomes, which is less precise because astigmatism can be induced by axis rotation. For this reason, vector analysis can generally describe the process of astigmatism correction, which focuses on both the magnitude and direction. Several vector-based methods [9, 20–23] that have been published have suggested that mathematic analysis could be used to describe the astigmatism. This study applied the method recommended by Alpins, which was regarded as the Alpins method of astigmatism analysis [9–11].

In terms of vector-based predictability, this study had a tendency to overcorrect the cylinder less than -0.50 D. There were similar results in the previous studies for the LASIK procedure [24, 25]. Undercorrection was observed as the cylinder increased, which was inconsistent with the results of MofE, CI and IOS in the present study. The MofE and CI indicated that slight overcorrection was observed when a preoperative cylinder was within -0.50 D and gradually changed to undercorrection as the cylinder increased. Even when the CI was nearly close to 1, there was still an approximately 20% deviation in the IOS from the ideal value, which should be 0. Possible reasons for a deviation in the IOS could be attributed to the axis rotation and decentration during the surgery [26]. Because SMILE surgery does not have eye-tracking, as we mentioned before, the direction of the astigmatism axis could be misaligned due to the shift of the pupil center. The pupil center shifted when the pupil diameter changed asymmetrically with different luminance [27]. In addition, the cyclotorsion from the upright to supine position could also induce errors in the treatment of astigmatism [28]. Alpins [11] noted that the treatment was misaligned by 30°, and the effect of correction was reduced by half while another half was canceled by the torque effect. When it came to 45°, there was no flattening effect at all. On the other hand, error might be observed particularly in the eyes with a large angle kappa preoperatively, and the correlation between the angle kappa and decentration should be confirmed by further investigation [29, 30]. The IOS value was highly correlated with the absolute AofE in this study, indicating the accuracy of axis correction could influence astigmatism treatment. Hjortdal et al. [31] indicated that undercorrection was predicted by increasing age and a steeper corneal curvature, which should also be considered before surgery. Recently, Ivarsen et al. [8] reported that the undercorrection was predicted by 13% per diopter of low myopic astigmatism and was 16% per diopter in high astigmatism, which was consistent with our results. In this study, the incision was performed at the 12 o’clock position; therefore, it was not supposed to induce the error of asymmetrical healing patterns or device-related errors. These results also suggested that to obtain the better results, accurate alignment was required. However, the exact reasons for inaccurate correction needed to be further investigated.

The effectiveness, safety and stability were also observed within the 1-year period after SMILE surgery in this study. It has been commonly observed that there is a regression in the eyes with excimer laser surgery, especially for high myopia [32]. However, the spherical diopters were almost fully corrected and stabilized within the 12-month follow-up in this study. Despite a tendency of undercorrection in astigmatic treatment, which should not be ignored, the mean postoperative astigmatism in vector form was -0.10 D at 1-year follow-up, which was much less than we had previously expected. Ang et al. [33] had also conducted a randomized controlled trial that indicated SMILE surgery was not worse than LASIK. Vestergaard et al. [3] reported that the mean cylinder magnitude was -0.41 ± 0.34 D at 3 months after SMILE surgery. For a longer follow-up in the current study, the mean cylinder magnitude was -0.20 ± 0.27 D at the 1-year period, which was gradually recovered with time. Similar results were also illustrated by Sekundo et al. [4] in the form of spherical equivalent and showed 38% gained one line, 4% gained two lines of CDVA with SMILE surgery. Recently, Kim et al. [34] found that age was also a predictor that influenced the visual acuity after surgery. Our results were comparable with theirs, indicating that the SMILE surgery had promising outcomes.

There were a few limitations in this study. Although the vector method is usually used to describe the refractive state of a paticular individual, instead of used to compare individuals, a comparative studies might be investigated in future. In addition, further assessment should be conducted to evaluate the correlation between astigmatism and large angle kappa, which might also play an important role in the treatment of astigmatism.

## Conclusions

Accordingly, SMILE surgery is a good choice for correcting low to moderate astigmatism especially in long-term results. The vector analysis is much more accurate for evaluating astigmatism correction than the previously used method of spherical equivalent. The tendency of undercorrection could remind surgeons to adjust nomograms for the correction of astigmatism with SMILE surgery. In addition, such undercorrection of astigmatism could possibly be influenced by several factors; for example, the attempted astigmatism correction preoperatively, the axis rotation during the surgery or the wound healing process postoperatively. Further investigations should be conducted to assess the treatment for correcting the higher astigmatism with SMILE surgery at longer follow-up.

## Abbreviations

SMILE: Small incision lenticule extraction
LASIK: Laser in-situ keratomileusis
FLEx: Femtosecond lenticule extraction
UDVA: Uncorrected distance visual acuity
CDVA: Corrected distance visual acuity
IOP: Intraocular pressure
TIA: Target induced astigmatism
SIA: Surgically induced astigmatism
DV: Difference vector
IOS: Index of success
CI: Correction index
AofE: Angle of error
MofE: Magnitude of error
SE: Spherical equivalent.
